# Supplementary material for: The Construction and Exploration of a Comprehensive MicroRNA Centered Regulatory Network in Foxtail Millet (Setaria italica L.)
Source: Front Plant Sci. 2022 May 6;13:848474. doi: 10.3389/fpls.2022.848474 (PMC9121102; doi:10.3389/fpls.2022.848474)
Supplement: Supplementary file 2 [file Data_Sheet_2.zip › Sit-miR319_Seita.2G033000.1_203_TPlot.pdf]

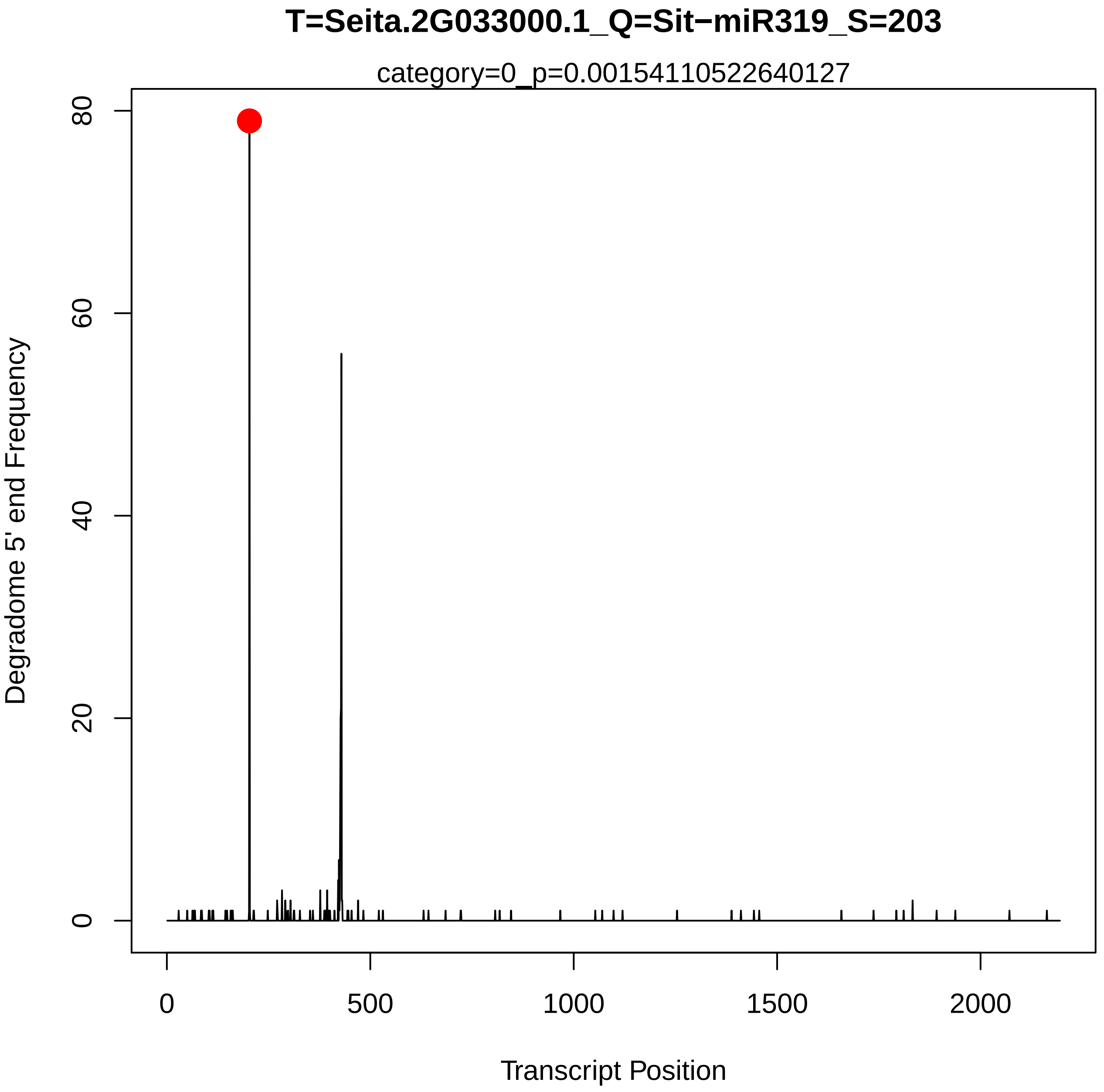
T=Seita.2G033000.1_Q=Sit−miR319_S=203
category=0_p=0.00154110522640127
Degradome 5' end Frequency
0
20
40
60
80
0
500
1000
1500
2000
Transcript Position
